# Combining protein and RNA quantification to evaluate promoter activity by using dual-color fluorescent reporting systems

**DOI:** 10.1042/BSR20211525

**Published:** 2021-09-10

**Authors:** Yan Peng, Xin Huang, Tianfang Huang, Feng Du, Xin Cui, Zhuo Tang

**Affiliations:** 1 Natural Products Research Center, Chengdu Institute of Biology, Chinese Academy of Sciences, Chengdu, Sichuan 610041, P.R. China; 2 Savaid Medical School, University of Chinese Academy of Sciences, Beijing 100049, P.R. China

**Keywords:** Broccoli, dual-color, fluorescent protein, Promoter, transcription, translation

## Abstract

Herein, Broccoli/*mCherry* and an *EGFP*/*mCherry* dual-color fluorescent reporting systems have been established to quantify the promoter activity at transcription and translation levels in eukaryotic cells. Based on those systems, four commonly used promoters (CMV and SV40 of Pol II and U6, H1 of Pol III) were accurately evaluated at both the transcriptional and translational levels by combining accurate protein and RNA quantification. Furthermore, we verified that Pol III promoters can induce proteins expression, and Pol II promoter can be applied to express RNA molecules with defined length by combining a self-cleaving ribozyme and an artificial poly(A) tail. The dual-color fluorescence reporting systems described here could play a significant role in evaluating other gene expression regulators for gene therapy.

## Introduction

Heterologous gene expression is critical to cell biology, particularly in gene therapy and cell therapy [1], through which exogenous gene encoding proteins or functional RNA molecules could express in the target organism. To improve the express efficiency of gene delivery and stability, both viral and non-viral vectors have been designed for the exogenous gene expression in eukaryotic cells [2,3]. The promoter on the vector is one of the most crucial elements, which leads to the initiation of transcription of a particular gene. In eukaryotes, RNA polymerases (Pol I, II, and III) are responsible for transcribing the distinct subsets of genes, synthesizing different classes of transcripts [4]. Pol II synthesizes mRNAs and some small nuclear (sn)RNAs, thus Pol II promoters are widely used for protein expression. Pol III uniquely transcribes small non-coding RNAs, including tRNAs, 5S rRNA, U6 snRNA, and so on [5]. Because Pol III promoters have defined transcription start and termination sites, they are mainly used for the expression of exogenous RNA molecular tools like ribozyme in gene knockdown and short hairpin RNA (shRNA) in RNAi applications [6]. With the completion of the Human Genome Project, intensive efforts have been devoted to find and develop different gene expression elements. Up to now, hundreds of diverse promoters have been discovering for exogenous gene expression. To accurately evaluate the activity of various promoters, the corresponding quantitative method is essential. Green fluorescent protein (GFP) and luciferase have quantified Pol II promoter activity at the translational level [7,8]. However, Pol III promoter activity has been analyzed indirectly by expressing RNA-silenced target proteins such as *GFP* and *p53* [9]. However, the currently used methods to evaluate promoter activity only focus on either the transcriptional or translational level. Several important questions have not yet been answered, such as whether the protein can express by Pol III promoters, whether the Pol II promoters can express non-coding RNA, and when the highest RNA and protein expression can be obtained by transient transfection.

Herein, we established two dual-color fluorescent reporting systems to accurately quantify the activity of promoters (Pol II and III) at the transcriptional and translational levels. Four commonly used promoters (CMV, SV40 of Pol II and U6, H1 of Pol III) have been used to evaluate RNA transcription and protein translation in eukaryotic cells. Based on this system, the transcriptional and translational efficiency of different promoters can be well evaluated qualitatively and quantitatively.

## Materials and methods

### Cells and reagents

High fidelity restriction endonucleases BamHI, EcoRI, HindIII, SalI, XhoI, and T4 ligase were purchased from NEB (New England Biolabs, MA, U.S.A.). Taq DNA polymerase and PFU DNA polymerase were purchased from TransGen (TransGen Biotech, Beijing, China). Bacterial strain Pro 5-α was purchased from Promega (WI, U.S.A.). Plasmids were prepared by AxyPrep™ Plasmid Miniprep Kit (Axygen, Corning, MA, U.S.A.). DNA products were purified by AxyPrep™ PCR Clean-up Kit (Axygen). Restriction enzyme digested-fragments were extracted by AxyPrep™ DNA Gel Extraction Kit (Axygen). DFHBI-1T was purchased from MCE (NJ, U.S.A.). DAPI was purchased from Solarbio (Beijing, China). HeLa and NIH-3T3 were gifts from Prof. Wang Fei’s laboratory (Chinese Academy of Sceinces, Chengdu, P.R. China).

### Plasmid construction

**Figure 1:**
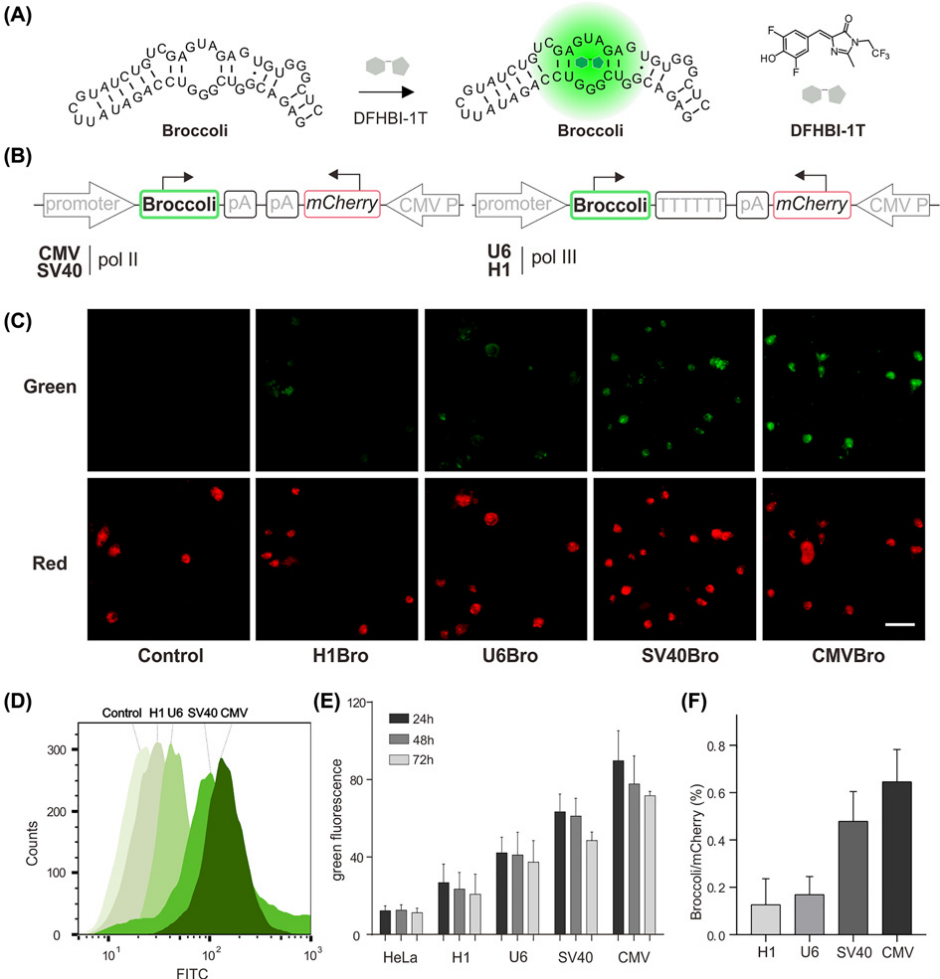
Analysis of different promoters at transcription level by Broccoli/*mCherry* dual-color fluorescence reporting system (**A**) The gray circle is DFHBI-1T which could be bonded and activated by Broccoli to produce green fluorescence. DFHBI-1T was treated at 20 μM for 10 min at room temperature. (**B**) Schematic diagrams of the genetic elements in plasmids. (**C**) The fluorescence imaging of HeLa cell after 24-h transfection. Green signals: Broccoli, Red signals: *mCherry*. (**D**) Flow-cytometry analysis of cells transfected with different vectors. (**E**) Flow cytometry histograms showing the green fluorescence intensity of Broccoli induced by different promoters. (**F**) Histograms showing the relative fluorescence intensity of Broccoli normalized to *mCherry.*

**Figure 2:**
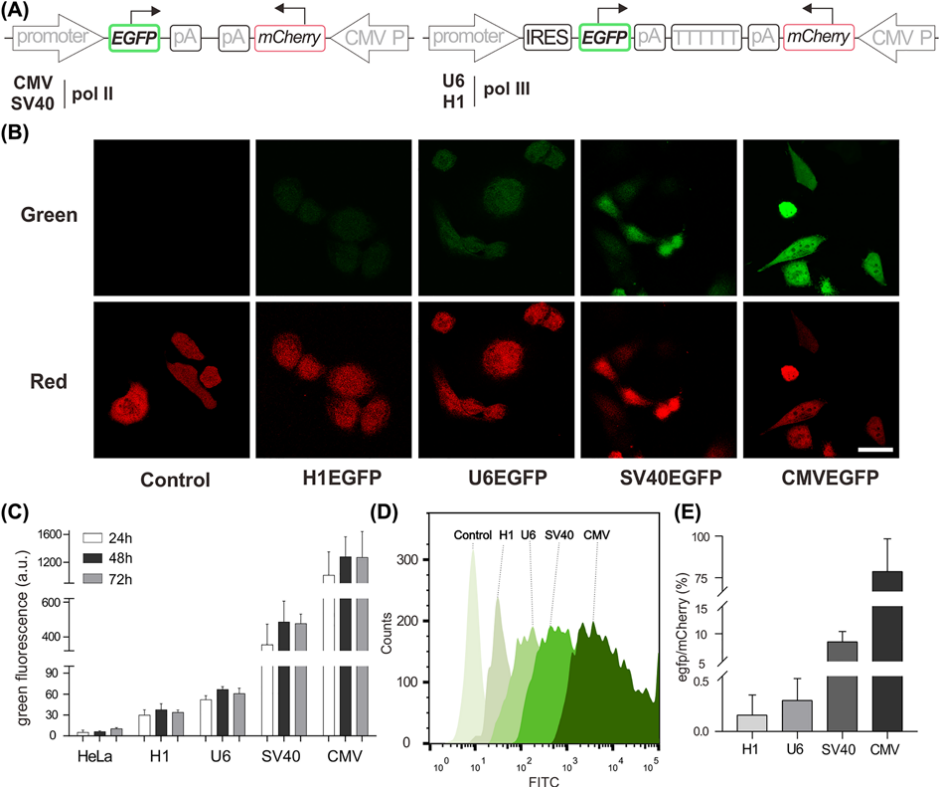
Analysis of different promoters at translational level by *eGFP*/*mCherry* dual-color fluorescence reporting system (**A**) Schematic diagrams of the genetic elements in plasmids. (**B**) The fluorescence imaging of HeLa cell after 44-h transfection. Green signals: *eGFP*, Red signals: *mCherry*. (**C**) Flow-cytometry analysis of cells transfected with different vectors. (**D**) Flow cytometry histograms showing the green fluorescence intensity of *eGFP* induced by different promoters. (**E**) Histograms showing the relative fluorescence intensity of *eGFP* normalized to *mCherry.*

**Figure 3:**
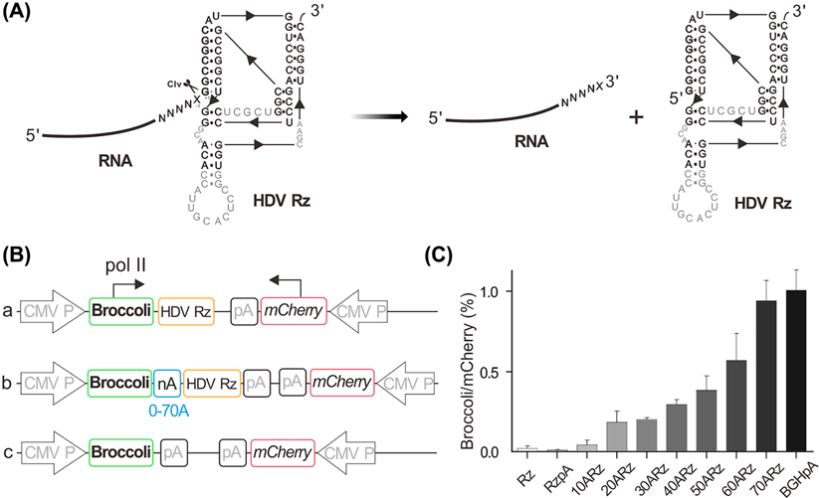
RNA Polymerase II transcription product was stabilized by Synthetic A-tail in high eukaryotic cell (**A**) Core elements are highlighted by region corresponding to the HDV ribozyme which can be self-cleavaged at the 5′-end; ‘X’ is any nucleotide except guanosine. (**B**) Schematic diagrams of the genetic elements in plasmids. (**C**) Relative fluorescence intensity of cells transfected with different vectors.

The vectors pmCherry-C1 (Invivogen), pSilencer 2.0-U6 (Ambion), psiRNA-h7SK hygro G1 (Invivogen), pEGFP-N1 (Invivogen), pcDNA 3.1+ (Invivogen) were used as the source for the two human Pol III promoters (U6 and H1), and the two virus Pol II promoters (CMV and SV40) (Supplementary Table S1). The four DNA fragments CMV promoter (GenBank accession number: AY446894.2) [10], SV40 promoter (GenBank accession number: J02400.1) [11], human U6 promoter (GenBank accession number: x07425) [12], human H1 promoter (GenBank accession number: X15624) [13], were synthesized by PCR. To generate all constructs (Figures 1B, 2A and 3B), the four promoters were inserted of the pmCherry-C1 vectors, Broccoli/EGFP were inserted behind the promoter, HDV/HDV+polyA were inserted behind Broccoli, the terminator signal was inserted behind Broccoli/EGFP and mCherry using the proper restriction enzyme sites (BamHI, SalI for CMV, SV40, U6 and H1 promoters; SalI, EcoRI for Broccoli/EGFP; EcoRI, HindIII for the terminator signal of CMV and SV40 promoter; EcoRI, HindIII for HDV/HDV+polyA of CMV promoter); XhoI, HindIII for the terminator signal of mCherry). The DNA fragments were synthesized by Integrated DNA Technology (IDT) and cloned into pmCherry-C1 vector by Gibson cloning according to the manufacturer’s instructions (New England Biolabs). The RzpA (CMV-Broccoli-HDVRz-pA), 10ARz (CMV-Broccoli-10A-HDVRz-pA), 20ARz (CMV-Broccoli-20A-HDVRz-pA), 30ARz (CMV-Broccoli-30A-HDVRz-pA), 40ARz (CMV-Broccoli-40A-HDVRz-pA), 50ARz (CMV-Broccoli-50A-HDVRz-pA), 60ARz (CMV-Broccoli-60A-HDVRz-pA), 70ARz (CMV-Broccoli-70A-HDVRz-pA) plasmid contain different lengths of artificial polyA in the HDV Rz front. The Rz (CMV-Broccoli-HDVRz) plasmid does not contain a polyA signal and the BGHpA (CMV-Broccoli-pA) plasmid contains a polyA signal. All constructs were verified by sequencing in Sangon (Shanghai, China). All vectors were verified by sequencing using the BigDye Terminator v1.1 Cycle Sequencing kit (ABI).

### Cell culture

HeLa cells and NIH-3T3 cells were cultured in DMEM (Life Technologies, Invitrogen, Carlsbad, CA, U.S.A.) supplemented with 10% fetal calf serum (FCS), penicillin (100 U/ml), and streptomycin (100 μg/ml). HeLa is a human cervical cancer cell line, and NIH-3T3 is a mouse embryonic fibroblasts cell line. Cells were trypsinized and seeded 1 day before transfection. Cells were cultured in a humidified incubator with 5% CO_2_ at 37°C.

### Western blot assay

Whole-cell protein extracts were prepared using RIPA lysis buffer (PC101, EpiZyme, China). Proteins were separated on a 10% SDS/PAGE gel and electroblotted on to an Immobilon-P membrane (Millipore). The membrane was blocked for 1 h at room temperature with 5% nonfat dry milk in Tris-buffered saline containing Tween 20 (TBST) buffer (20 mM Tris/HCl [pH 7.5], 150 mM NaCl, and 0.1% Tween 20), then incubated with the primary antibody overnight at 4°C, washed with TBST buffer, and incubated with HRP-conjugated secondary antibody for 1.5 h at room temperature. After being washed with TBST buffer, blots were developed with SuperSignal West Pico or Femto chemiluminescent substrate (Pierce) or ECL Plus Western blotting detection reagents (GE Healthcare). Antibodies used were rabbit monoclonal to α-tubulin (ab179484, Abcam, Cambridge, U.K.), rabbit monoclonal to EGFP (ab184601, Abcam, Cambridge, U.K.). Proteins recognized by the antibodies were detected by ImageQuant LAS 500 (GE, CT, U.S.A.) using HRP-conjugated Goat Anti-Rabbit secondary antibody (BBI, Sangon Biotech, China).

### Dual-color reporter assay

All constructs were transfected into HeLa and NIH-3T3 cells with 1000 ng plasmid using Lipofectamine 2000 (Invitrogen) according to manufacturer’s instructions. One-day, two-day, and three-day post-transfection, Broccoli and *EGFP* activity was measured by flow cytometry (FCM) and Confocal Laser Scanning Microscope (CLSM). The results were corrected by *mCherry* fluorescence as described previously.

### Dual-color reporter system using FCM

Cells were trypsinized and seeded 1 day before transfection. Then dual-color reporter constructs were transfected into HeLa and NIH-3T3 cell lines. After 1-, 2-, and 3 days post-transfection, cell pellets were resuspended by 1× PBS and analyzed by FCM (green fluorescence, excitation at 480 nm, emission at 510 nm; mCherry fluorescence, excitation 561 nm, emission 610 nm). *EGFP* or Broccoli was co-expressed with *mCherry* which was used as an internal reference. In Broccoli expression system, 20 μM DFHBI-1T is added for 10 min at 37°C and then washed three times with 1× PBS for flow analysis. Only cells with normal *mCherry* fluorescence would be analyzed by FCM.

### Observation of two-color fluorescence using CLSM

For fluorescence microscopy, live cells were analyzed on an Eclipse Ts2R inverted microscope (Nikon, Tokyo, Japan) and images were acquired using NIS-Elements imaging software (Nikon). Exposure time and gain are kept constant for all acquired images within an experimental series. For confocal images, cells were analyzed on a Leica SP8 microscope (Leica Microsystems, Wetzlar, Germany) and images were acquired using LAS X software (Leica Microsystems). Images were processed and analyzed using the Fiji distribution of ImageJ. Cells were trypsinized and seeded 1 day before transfection. Then dual-color reporter constructs were transfected into HeLa and NIH-3T3 cell lines. Then transfected cells were inoculated into the glass-bottom dish. After 1-, 2-, and 3-day post-transfection, cells were cleaned by 1× PBS and analyzed by CLSM (EGFP fluorescence, excitation at 480 nm, emission at 510 nm; Broccoli fluorescence, excitation at 469 nm, emission at 501 nm; mCherry fluorescence, excitation 589 nm, emission 610 nm). In brief, cells were lysed in 125 mM KCl, 5 mM MgCl_2_, 40 mM HEPES pH 7.4 and 20 μM DFHBI-1T. *EGFP* or Broccoli was co-expressed with mCherry. In the Broccoli expression system, 20 μM DFHBI-1T was added and incubated for 10 min at 37°C and then washed three times with 1× PBS for CLSM. Aside from the FCM analysis, the major difference is that mammalian cells are generally dimmer and thus it is harder to find bright fluorescent cells. If the photobleaching happened, cells should be allowed to rest in the dark before imaging again and add new fluorescein. Also, mammalian cells are sensitive to the temperature and media pH, so experiments should be performed at 37°C with CO_2_ level of 5% (or in a buffered media).

## Results and discussion

### Analysis of different promoters at transcriptional level by Broccoli/mCherry dual-color fluorescence reporting system

Once transferred into the cell, the exogenous gene must be transcribed first by the corresponding RNA polymerase depending on the promoter on it. In 2011, Jafrey and colleagues [14] developed fluorescent light-up RNA aptamers (FLAPs) known as mimics of GFPs [14,15]. Recently, Broccoli aptamer was discovered by Jaffrey and colleagues, which has short RNA sequences and higher brightness through binding and activating the fluorophore (DFHBI-1T) in mammalian cells (Supplementary Figures S1–S3) [16]. Subsequently, its applications as sensors *in vivo* have been increasingly reported [17]. Therefore, the Broccoli aptamer was used as an RNA mimic of GFP to measure the promoter activity here (Figure 1A and Supplementary Figure S4). In order to obtain more accurate quantitative results, *mCherry* with CMV promoter was introduced as an internal reference to normalize the Broccoli RNA expression initiated by different promoters (CMV, SV40, U6, and H1 as shown in Figure 1B). After transient transfection of HeLa cell with the corresponding recombinant plasmids, we can directly observe the green fluorescence of Broccoli with different intensity in presence of DFHBI-1T and consistent red fluorescence of *mCherry* in various groups of cells by using CLSM (Figure 1C and Supplementary Figure S5). To get quantifiable results of RNA expression by various promoters, those cells were analyzed by using FCM (Supplementary Figure S6). As shown in Figure 1D, the order of activity of the four promoters is CMV > SV40 > U6 > H1 according to the fluorescent intensity of transcribed RNA. To explore the relationship between the amounts of RNA expressed by transient transfection with time, we have recorded the green fluorescence intensity of Broccoli for 72 h and found that the maximum RNA expression was reached 24 h after transfection for all four promoters (Figure 1E). More accurate results at 24 h were obtained by normalizing the RNA expression with *mCherry* reference, revealing that the Broccoli RNA transcribed by Pol II with CMV promoter is three-times as much RNA transcribed by Pol III U6 promoter (Figure 1F). These results showed that the Pol II promoters have a higher transcriptional ability than Pol III promoters, providing a significant potential in exogenous RNA expression.

### Analysis at the transcriptional level

(A) The gray circle is DFHBI-1T which could be bonded and activated by Broccoli to produce green fluorescence. DFHBI-1T was treated at 20 μM for 10 min at room temperature. (B) Schematic diagrams of the genetic elements in plasmids. (C) The fluorescence imaging of HeLa cell after 24-h transfection. Green signals: Broccoli, Red signals: *mCherry*. (D) FCM analysis of cells transfected with different vectors. (E) FCM histograms showing the green fluorescence intensity of Broccoli induced by different promoters. (F) Histograms showing the relative fluorescence intensity of Broccoli normalized to *mCherry.*

### Analysis of different promoters at translational level by *eGFP*/*mCherry* dual-color fluorescence reporting system

After evaluation of the activity of four promoters at the transcription level, we sought to quantify its expression activity at the translational level. In previous work, luciferases have been applied to evaluate promoter expression efficiency, but this method relies on the indirect determination of the products of catalyzed chemiluminescence reaction products [18,19]. Fluorescent proteins could be a better choice because they can directly reflect the protein expression through fluorescent intensity. In 2006, Rumi and colleagues used *EGFP* to quantify the activity of Pol II promoters [20], whose expression could be affected by the transfection efficiency and inconsistent cell growth. Therefore, we constructed a new dual-color fluorescent reporting system, in which *EGFP* was used as the reporter gene to reveal the protein expression with the different promoters, respectively, while *mCherry* with CMV promoter was added into the vector as a reference (Figure 2A). HeLa cells were transiently transfected with corresponding plasmids, and according to the result of CLSM (Figure 2B and Supplementary Figure S7), the transfected cells with Pol II promoters (CMV and SV40) produced high-brightness green fluorescent signals as expected 48 h after transfection. It has been reported that the Pol III promoters could mediate *luciferase* reporter gene expression by recruiting Pol II and a Pol II termination signal AAUAAA at 3′-end is required for the synthesis of poly(A) that is critical to the following translation [8,21]. Therefore, the IRES element that is essential for translation initiation was inserted to the 5′ *EGFP* sequence, and a polyA signal (62-bp) was added in between *EGFP* and Pol III termination signal TTTTTT (Figure 2A and Supplementary Table S2) [22,23]. Intriguingly, the expression of green fluorescence was also observed in the cells transfected with the vectors with Pol III promoter (U6 and H1, Figure 2B–D). Those results were further verified with FCM, revealing that the protein expression reaching a peak 48 h after transfection, and the order of activity of the four promoters at the translational level was CMV > SV40 >> U6 > H1 (Figure 2C,D and Supplementary Figure S8). The accurate quantification of four promoters was obtained after normalizing the protein expression with *mCherry* reference, showing that *egfp* proteins induced by Pol II promoter are ∼20 times more proteins induced by Pol III promoter (Figure 2E). Moreover, the *EGFP* expressions induced by different promoters were measured by immunoblotting (Supplementary Figure S9). The results demonstrated that the expression efficiency of the CMV promoter was the highest and the Pol III promoters (U6 and H1) were also able to induce the expression of *EGFP*, which is consistent with the observed results of the *EGFP*/*mCherry* dual-color fluorescent reporting system.

### Analysis at the translational level

(A) Schematic diagrams of the genetic elements in plasmids. (B) The fluorescence imaging of HeLa cells after 44-h transfection. Green signals: *eGFP*, Red signals: *mCherry*. (C) FCM analysis of cells transfected with different vectors. (D) FCM histograms showing the green fluorescence intensity of *eGFP* induced by different promoters. (E) Histograms showing the relative fluorescence intensity of *eGFP* normalized to *mCherry.*

### RNA polymerase II transcription product were stabilized by synthetic A-tail in high eukaryotic cell

Besides HeLa cells, we used murine cells (NIH-3T3) to evaluate all four promoters’ transcriptional and translational efficiency, and the results were consistent with the human cells (Supplementary Figures S5 and S7). Our obtained results indicated that the CMV promoter of Pol II afforded the highest activity at both the transcriptional and translational levels among all four promoters. But it has been rarely applied in the expression of RNA molecular tools. The greatest weakness of using Pol II promoter to transcribe functional RNA molecules is that a poly(A) tail [24,25] of (150–250 nt) will be added through polyadenylation after the cleavage of termination signal AAUAAA in the eukaryotic cell [26], yielding the RNA transcript with changeable length [27].

Therefore a self-cleaving HDV ribozyme (HDVRz) that can (self-)cleave at its 5′-end (Figure 3A) was inserted behind the 3′ end of the Broccoli sequence to cleave the termination signal AAUAAA sequence (Figure 3B), thus obtaining an RNA transcript with fixed length after the transcription by Pol II. However, the introduced ribozyme resulted in the disappearance of the green fluorescence (Figure 3C and Supplementary Figure S10), revealing that the poly(A) tail has an essential role in RNA stability. Therefore, an artificial poly(A) tail was added between Broccoli and HDVRz sequence to ensure RNA stability. We systematically investigated the difference of tail length on RNA stability (from 10 to 70 nt, Figure 3B). With the increase in tail length, the fluorescence of cleaved Broccoli RNA was restoring gradually, and the RNA expression was reached 94% of original expression with a 70-nt artificial poly(A) tail, the fluorescence intensity almost unchanged with the increase of the length of artificial poly(A) tail (Figure 3C and Supplementary Figure S10). Based on those results, the RNA molecules of precise length could be expressed with Pol II promoter through adding a self-cleaving ribozyme and an artificial poly(A) tail.

### Investigation of synthetic A-tail

(A) Core elements are highlighted by region corresponding to the HDVRz which can be self-cleaved at the 5′-end; ‘X’ is any nucleotide except guanosine. (B) Schematic diagrams of the genetic elements in plasmids. (C) Relative fluorescence intensity of cells transfected with different vectors.

## Conclusion

In summary, a Broccoli/*mCherry* and an *EGFP*/*mCherry* dual-color fluorescent reporting system have been constructed to accurately quantify the promoter activity at transcriptional and translational levels in eukaryotic cells. Based on those systems, four commonly used promoters were evaluated by combining accurate protein and RNA quantification, and we drew the following conclusions: (i) both Pol II and Pol III promoters can induce the protein expression, and the order of activity of the four promoters at the translational level was CMV > SV40 >> U6 > H1, showing the Pol II promoters are ten-times more efficient than Pol III promoters; (ii) but, at the transcriptional level, the efficiency differences between them are not so significant, only several times; (iii) the highest RNA expression was reached 24 h after transfection for all four promoters, while the protein expression reached the highest expression 48 h after transfection.

Furthermore, through the introduction of a self-cleaving ribozyme, Pol II promoters can be applied to the length-controllable expression of the RNA molecule, their expression can be modulated by adding an artificial poly(A) tail. The present study had proven that both Pol II and Pol III promoters could be used to either heterogenous RNA or protein expression, regardless of their conventional roles. Therefore, Pol II promoters can be used for the expression of different functional RNA molecules, including shRNA in RNAi applications, guide RNA (gRNA) in CRISPR-Cas9 genome-editing platforms, ribozyme in gene therapy applications, and so on. Both Pol II and Pol III promoters have been shown to be capable of protein expression, thus providing more options for the expression of therapeutic proteins with different functions. Moreover, we believed that the dual-color fluorescence reporting system described here could play a significant role in evaluating other gene expression regulators for gene therapy.

## Supplementary Material

Supplementary Figures S1-S2 and Tables S1-S11

## Data Availability

Data are contained within the article.

## Abbreviations

CLSM: confocal laser scanning microscope
DAPI: 4',6-diamidino-2-phenylindole
DFHBI-1T: (Z)-4-(3,5-difluoro-4-hydroxybenzylidene)-2-methyl-1-(2,2,2-trifluoroethyl)-1H-imidazol-5(4 H)-one
FCM: flow cytometry
FLAPs: fluorescent light-up RNA aptamers
GFP: green fluorescent protein
gRNA: guide RNA
HDV: hepatitis D virus
HDVRz: HDV ribozyme
HRP: Horseradish Peroxidase
RNAi: RNA interference
shRNA: short hairpin RNA
TBST: Tris-buffered saline containing Tween 20
