# Supplementary material for: Combining protein and RNA quantification to evaluate promoter activity by using dual-color fluorescent reporting systems
Source: Biosci Rep. 2021 Sep 10;41(9):BSR20211525. doi: 10.1042/BSR20211525 (PMC8433482; doi:10.1042/BSR20211525)
Supplement: Supplementary Figures S1-S2 and Tables S1-S11 [file BSR-2021-1525_supp.pdf]

Supplementary Material

Combining Protein and RNA Quantification to Evaluate Promoter Activity by Using Dual-color Fluorescent Reporting Systems

Yan Peng[a,b], Xin Huang[a], Tianfang Huang[a], Feng Du[a], Xin Cui[a] and Zhuo Tang[a]*

[a]Natural Products Research Center, Chengdu Institute of Biology, Chinese Academy of Sciences, Chengdu, Sichuan, 610041, P.R. China. E-mail: tangzhuo@cib.ac.cn.

[b]University of Chinese Academy of Sciences, Beijing, 100049, P.R. China.

Abstract: Herein, a Broccoli/*mCherry* and an *EGFP*/*mCherry* dual-color fluorescent reporting systems have been established to quantify the promoter activity at transcription and translation level in eukaryotic cells. Based on those systems, four commonly used promoters (CMV and SV40 of Pol II and U6, H1 of Pol III) were accurately evaluated at both the transcriptional and translational levels by combining accurate protein and RNA quantification. Furthermore, we verified that Pol III promoters can induce proteins expression, and Pol II promoter can be applied to express RNA molecules with defined length by combining a self-cleaving ribozyme and an artificial poly(A) tail. The dual-color fluorescence reporting systems described here could play a significant role in evaluating other gene expression regulators for gene therapy.

Keywords

Promoter; transcription; translation; dual-color; Broccoli; fluorescent protein;

MATERIALS AND METHODS **3**
Cells and reagents 3
Plasmid Construction 3
Cell Culture 3
Western Blot Assay 4
Dual-Color Reporter Assay 4
Dual-color reporter system using flow cytometry (FCM) 4
Observation of two-color fluorescence using Confocal Laser Scanning Microscope (CLSM) 4
The sequence used in the experiment **5**
Table S1. The cDNA sequence of the following human DNA was obtained from NCBI. 5
Table S2. The construction of plasmid sequence information. 5
Results and Discussion **6**
Supplementary Figure S1 6
Supplementary Figure S2 6
Supplementary Figure S3 7
Supplementary Figure S4 7
Supplementary Figure S5 8
Supplementary Figure S6 8
Supplementary Figure S7 9
Supplementary Figure S8 9
Supplementary Figure S9 10
Supplementary Figure S10 10
Supplementary Figure S11 （uncropped Western blot images） 11
Reference **11**

MATERIALS AND METHODS

Cells and reagents

High fidelity restriction endonuclease BamHI, EcoRI, HindIII, SalI, XhoI, and T4 ligase were purchased from NEB (New England Biolabs, MA, USA). Taq DNA polymerase and PFU DNA polymerase were purchased from TransGen (TransGen Biotech, Beijing, China). Bacterial Strain Pro 5-alpha was purchased from Promega (Promega, WI, USA). Plasmids were prepared by AxyPrepTM Plasmid Miniprep Kit (Axygen, Corning, MA, USA). DNA products were purified by AxyPrepTM PCR clean-up Kit (Axygen). Restriction enzyme digested fragments were extracted by AxyPrepTM DNA Gel Extraction Kit (Axygen). DFHBI-1T was purchased from MCE (MCE, NJ, USA). DAPI was purchased from Solarbio (Solarbio, Beijing, China). HeLa and NIH-3T3 were gifted from Pr. WangFei's laboratory.

Plasmid Construction

The vectors pmCherry-C1 (Invivogen), pSilencer 2.0-U6 (Ambion), psiRNA-h7SK hygro G1 (Invivogen), pEGFP-N1 (Invivogen), pcDNA 3.1+ (Invivogen) were used as the source for the two human Pol III promoters (U6 and H1), and the two virus Pol II promoters (CMV and SV40) (Table S1). The four DNA fragments CMV promoter (GenBank accession number: AY446894.2),(1) SV40 promoter (GenBank accession number: J02400.1),[(2)] human U6 promoter (GenBank accession number:x07425),(3) human H1 promoter (GenBank accession number: X15624),(4) were synthesized by PCR reaction. To generate all constructs (Figures 1B, Figures 2A and Figures 3B), the four promoters were inserted of the pmCherry-C1 vectors, Broccoli/EGFP were inserted behind the promoter, HDV/HDV+polyA were inserted behind Broccoli, the terminator signal was inserted behind Broccoli/EGFP and mCherry using the proper restriction enzyme sites (BamHI, SalI for CMV, SV40, U6 and H1 promoters; SalI, EcoRI for Broccoli/EGFP; EcoRI, HindIII for the terminator signal of CMV and SV40 promoter; EcoRI, HindIII for HDV/HDV+polyA of CMV promoter); XhoI, HindIII for the terminator signal of mCherry). The DNA fragments were synthesized by Integrated DNA Technology (IDT) and cloned into the pmCherry-C1 vector by Gibson cloning according to the manufactures' instructions (New England Biolabs). The RzpA (CMV-Broccoli-HDVRz-pA), 10ARz (CMV-Broccoli-10A-HDVRz-pA), 20ARz (CMV-Broccoli-20A-HDVRz-pA), 30ARz (CMV-Broccoli-30A-HDVRz-pA), 40ARz (CMV-Broccoli-40A-HDVRz-pA), 50ARz (CMV-Broccoli-50A-HDVRz-pA), 60ARz (CMV-Broccoli-60A-HDVRz-pA), 70ARz (CMV-Broccoli-70A-HDVRz-pA) plasmid contain different lengths of artificial polyA in the HDV Rz front. The Rz (CMV-Broccoli-HDVRz) plasmid does not contain a polyA signal and the BGHpA (CMV-Broccoli-pA) plasmid contains a polyA signal. All constructs were verified by sequencing in Sangon (Sangon, Shanghai, China). All vectors were verified by sequencing using the BigDye Terminator v1.1 Cycle Sequencing kit (ABI).

Cell Culture

HeLa cells and NIH-3T3 cells were cultured in DMEM (Life Technologies, Invitrogen, Carlsbad, CA, USA) supplemented with 10% fetal calf serum (FCS), penicillin (100 U/mL), and streptomycin (100 ug/mL). HeLa is a human cervical cancer cell line, and NIH-3T3 is a mouse embryonic fibroblasts cell line. Cells were trypsinized and seeded 1 day before transfection. Cells were cultured in a humidified incubator with 5% CO_2 at 37℃.

Western Blot Assay

Whole-cell protein extracts were prepared using RIPA lysis buffer (PC101, EpiZyme, CHINA). Proteins were separated on a 10% SDS-PAGE gel and electroblotted onto an Immobilon-P membrane (Millipore). The membrane was blocked for 1 h at room temperature with 5% nonfat dry milk in Tris-buffered saline containing Tween 20 (TBST) buffer (20 mM Tris-HCl [pH 7.5], 150 mM NaCl, and 0.1% Tween 20), then incubated with the primary antibody overnight at 4°C, washed with TBST buffer, and incubated with HRP-conjugated secondary antibody for 1.5 h at room temperature. After being washed with TBST buffer, blots were developed with SuperSignal West Pico or Femto chemiluminescent substrate (Pierce) or ECL Plus Western blotting detection reagents (GE Healthcare). Antibodies used were rabbit monoclonal to α-tubulin (ab179484, Abcam, Cambridge, UK), rabbit monoclonal to EGFP (ab184601, Abcam, Cambridge, UK). Proteins recognized by the antibodies were detected by ImageQuant LAS 500 (GE, CT, USA) using HRP-conjugated Goat Anti-Rabbit secondary antibody (BBI, Sangon Biotech, CHINA).

Dual-Color Reporter Assay

All constructs were transfected into HeLa and NIH-3T3 cells with 1000ng plasmid using lipofectamine 3000 (Invitrogen) according to manufactures' instructions. One day, two-day and three-day post-transfection, Broccoli and *EGFP* activity was measured by flow cytometry and Confocal Laser Scanning Microscope. The results were relatively corrected by *mCherry* fluorescence as described previously.

Dual-color reporter system using flow cytometry (FCM)

Cells were trypsinized and seeded 1 day before transfection. Then dual-color reporter constructs were transfected into HeLa and NIH-3T3 cell lines with 1000 ng plasmid using lipofectamine 3000 (Invitrogen) according to the manufactures' instructions. After one day, two-day, and three-day post-transfection, Cell pellets were resuspended by 1*PBS and analyzed by FCM (Green fluorescence, excitation at 480 nm, emission at 510 nm; Red fluorescence, excitation 561 nm, emission 610 nm). *EGFP* or Broccoli was co-expressed with *mCherry* which was used as an internal reference. In Broccoli expression system, 20uM DFHBI-1T is added for 10 minutes at 37℃ and then washed three times with 1*PBS for flow analysis. Only cells with normal *mCherry* fluorescence would be analyzed by FCM.

Observation of two-color fluorescence using Confocal Laser Scanning Microscope (CLSM)

For fluorescence microscopy, live cells were analyzed on an Eclipse Ts2R inverted microscope (Nikon, Tokyo, Japan) and images were acquired using NIS-Elements imaging software (Nikon). Exposure time and gain are kept constant for all acquired images within an experimental series. For confocal images, cells were analyzed on a Leica SP8 microscope (Leica Microsystems, Wetzlar, Germany) and images were acquired using LAS X software (Leica Microsystems). Images were processed and analyzed using the Fiji distribution of ImageJ. Cells were trypsinized and seeded one day before transfection. Then dual-color reporter constructs were transfected into HeLa and NIH-3T3 cell lines. Then transfected cells were inoculated into the glass-bottom dish. After one day, two-day, and three-day post-transfection, Cells were cleaned by 1 x PBS and analyzed by CLSM (EGFP fluorescence, excitation at 480 nm, emission at 510 nm; Broccoli fluorescence, excitation at 469 nm, emission at 501 nm; mCherry fluorescence, excitation 589 nm, emission 610 nm). In brief, cells were lysed in 125mM KCl, 5mM $MgCl_2$, 40mM HEPES PH7.4 and 20uM DFHBI-1T. *EGFP* or Broccoli was co-expressed with *mCherry*. In the Broccoli expression system, 20uM DFHBI-1T was added and incubated for 10 minutes at 37℃ and then washed three times with 1 x PBS for CLSM. Aside from the FCM analysis, the major difference is that mammalian cells are generally dimmer and thus it is harder

to find bright fluorescent cells. If the photobleaching happened, cells should be allowed to rest in the dark before imaging again and add new fluorescein. Also, mammalian cells are sensitive to the temperature and media pH, so experiments should be performed at 37℃ with CO_2 level of 5% (or in a buffered media).

The sequence used in the experiment

Table S1. The cDNA sequence of the following human DNA was obtained from NCBI.

CMV promoter	NCBI Reference Sequence: AY446894.2
SV40 promoter	NCBI Reference Sequence: J02400.1
U6 promoter	NCBI Reference Sequence: x07425
H1 promoter	NCBI Reference Sequence: X15624

Table S2. The construction of plasmid sequence information.

Broccoli	GAGACGGTCGGGTCCAGATATTCGTATCTGTCGAGTAGAGTGTGGGCTCC
IRES	ACGTTACTGGCCGAAGCCGCTTGGAATAAGGCCGGTGTGCGTTTGTCTATATGTTATTTTCCACCATATTGCC<br>GTCTTTTGGCAATGTGAGGGCCCGGAAACCTGGCCCTGTCTTCTTGACGAGCATTCCTAGGGGTCTTTCCCCT<br>CTCGCCAAAGGAATGCAAGGTCTGTTGAATGTCGTGAAGGAAGCAGTTCCTCTGGAAGCTTCTTGAAGACAA<br>ACAACGTCTGTAGCGACCCTTTGCAGGCAGCGGAACCCCCCACCTGGCAACAGGTGCCTCTGCGGCCAAAAG<br>CCACGTGTATAAGATACACCTGCAAAGGCGGCACAACCCCAGTGCCACGTTGTGAGTTGGATAGTTGTGGAA<br>AGAGTCAAATGGCTCTCCTCAAGCGTATTCAACAAGGGGCTGAAGGATGCCCAGAAGGTACCCCATTGTATG<br>GGATCTGATCTGGGGCCTCGGTGCACATGCTTTACATGTGTTTAGTCGAGGTTAAAAAAACGTCTAGGCCCCC<br>CGAACCACGGGGACGTGGTTTTCCTTTGAAAAACACGATGATAA
Poly(A) signal	AATAAAGGATCTTTTATTTTCATTGGATCTGTGTGTTGGTTTTTTGTATGCGGCCGCTAGCT
HDV Rz	GGCCGGCATGGTCCCAGCCTCCTCGCTGGCGCCGGCTGGGCAACATGCTTCGGCATGGCGAATGGGAC

Results and Discussion

Supplementary Figure S1

Excitation and emission spectra of Broccoli

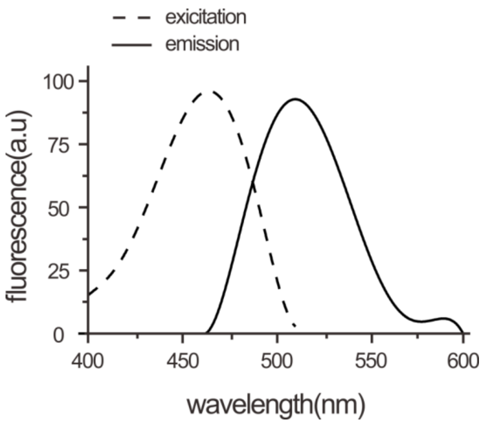

Fig S1. *In vitro* comparison of DFHBI-1T complex with Broccoli. Spectra were measured using 1*u*M RNA and 10*u*M DFHBI-1T.

Supplementary Figure S2

Absorbance spectra of DFHBI-1T alone and in complex with Broccoli

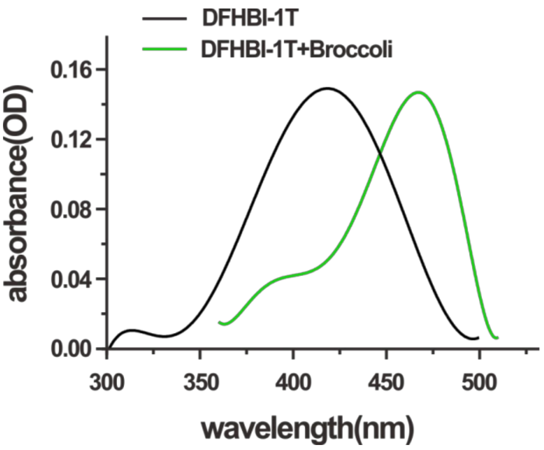

Fig S2. Absorbance spectra of DFHBI-1T alone and in complex with Broccoli show a red-shift similar to that previously observed for Broccoli-DFHBI-1T(5); here, 1*u*M RNA was preincubated with 5*u*M fluorophore, and the spectrum was compared to that of the fluorophore alone.

Supplementary Figure S3

Dissociation constant of Broccoli

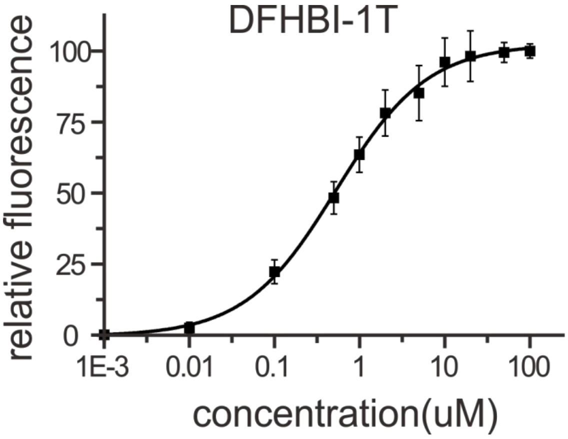

Fig S3. To calculate the dissociation constant we performed a titration of 50nM RNA with increasing concentration of DFHBI-1T and then fitted the resulting data points using the Hill equation as described previously(6). Error bars indicate standard deviations (n=3).

Supplementary Figure S4

Strategies to Solve Weak Fluorescence Signals of Broccoli

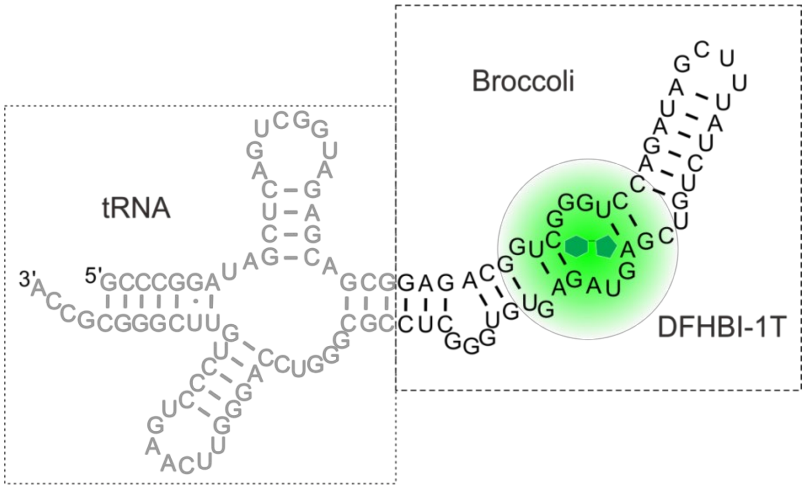

Fig S4. Schematic diagram of Broccoli combined with tRNA scaffold. The tRNA scaffold added to enhance the stability of the Broccoli structure in eukaryotic cells. The Broccoli aptamer can bind with small molecule (DFHBI-1T), which is a mimic of the chromophore of green fluorescent protein, the tRNA scaffold were added to enhance the stability of the Broccoli structure in eukaryotic cells.

Supplementary Figure S5

RNA Quantification to Evaluate Promoter Activity in NIH-3T3 cell

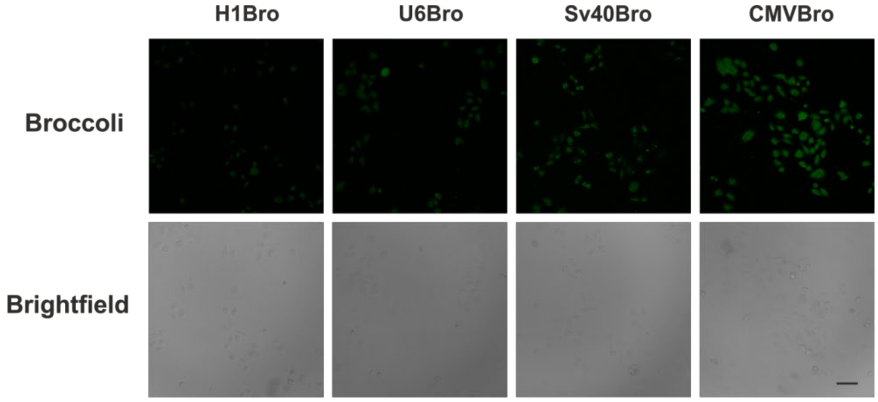

Fig S5. CLSM images of NIH-3T3 cell after 10 min incubation with 20uM DFHBI-1T. From left to right CLSM pictures presented the green fluorescence of NIH-3T3 transformed with H1Bro, U6Bro, SV40Bro and CMVBro plasmid, showing the green fluorescence intensity was increasing orderly. The green fluorescence observed by CLSM 24 hours later.

Supplementary Figure S6

RNA Quantification to Evaluate Promoter Activity by Using Dual-color Fluorescent Reporting Systems in HeLa cells

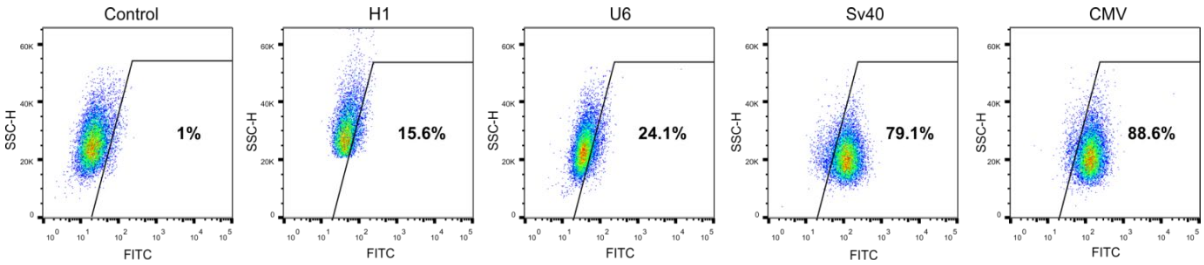

Fig S6. Twenty-four hours after transfection of HeLa cells with dual-color fluorescent expression plasmid, 20,000 cells were collected and analyzed by flow cytometry using 20uM DFHBI-1T.

Supplementary Figure S7

Protein Quantification to Evaluate Promoter Activity in NIH-3T3 cell

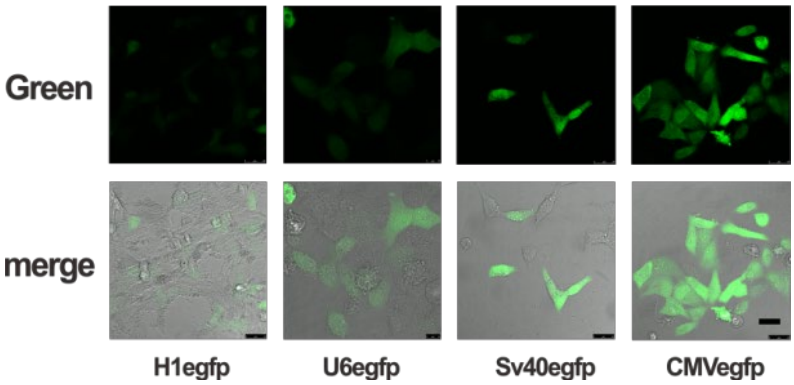

Fig S7. CLSM images of green fluorescence in NIH-3T3 cell after 48 hours post-transfection. From left to right CLSM pictures presented the green fluorescence of NIH-3T3 transformed with H1GFP, U6GFP, SV40GFP and CMVGFP plasmids, showing the green fluorescence intensity was increasing orderly. The green fluorescence observed by CLSM.

Supplementary Figure S8

Protein Quantification to Evaluate Promoter Activity by Using Dual-color Fluorescent Reporting Systems in HeLa cells

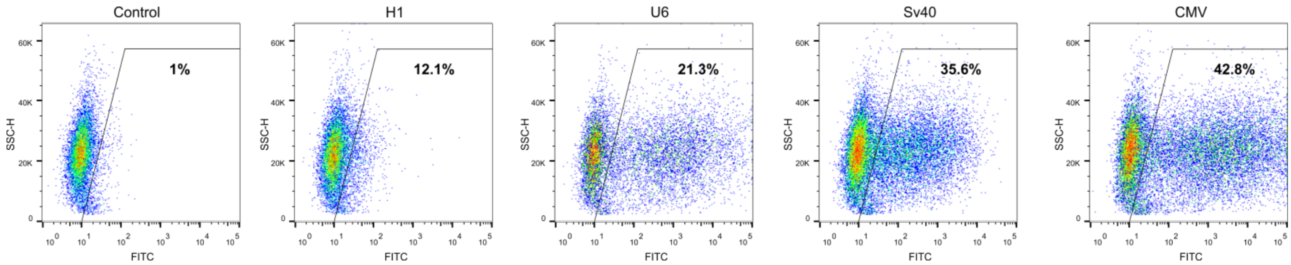

Fig S8. Forty-eight hours after transfection of HeLa cells with two-color fluorescent expression plasmid, 20,000 cells were collected and analyzed by flow cytometry.

Supplementary Figure S9

Protein Quantification to Evaluate Promoter Activity by Using Western Blot

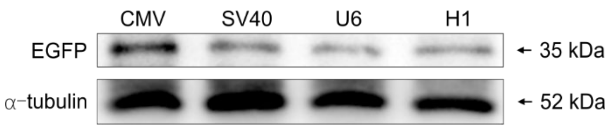

Fig S9. HeLa cells were transfected with different plasmids for 48 hours and proteins were extracted for Western blot assay and blotted for EGFP, α-tubulin.

Supplementary Figure S10

CMV promoter of Pol II artificial poly (A) tail length on RNA stability

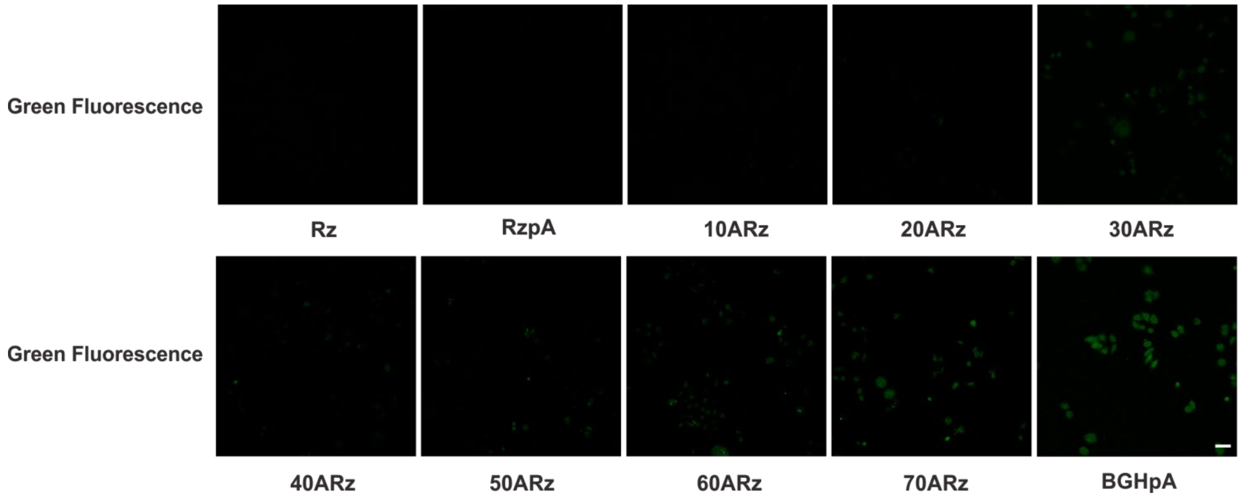

Fig S10. CLSM images of HeLa cells after 10 min incubation with 20uM DFHBI-1T. Rz means no pA signal sequence, BGHpA means pA signal sequence. Rz (CMV-Broccoli-HDVRz), RzpA (CMV-Broccoli-HDVRz-pA), 10ARz (CMV-Broccoli-10A-HDVRz-pA), 20ARz (CMV-Broccoli-20A-HDVRz-pA), 30ARz (CMV-Broccoli-30A-HDVRz-pA), 40ARz (CMV-Broccoli-40A-HDVRz-pA), 50ARz (CMV-Broccoli-50A-HDVRz-pA), 60ARz (CMV-Broccoli-60A-HDVRz-pA), 70ARz (CMV-Broccoli-70A-HDVRz-pA), indicate that the length of artificially synthesized polyA is 0, 10A, 20A, 30A, 40A, 50A, 60A, 70A, BGHpA (CMV-Broccoli-pA). We can see that the longer the length of the synthetic polyA tail, the brighter the fluorescence in the living cells. The green fluorescence observed by CLSM 24 hours later.

Supplementary Figure S11 （uncropped Western blot images）

Supplementary Figure S11: Fig S9_uncropped images

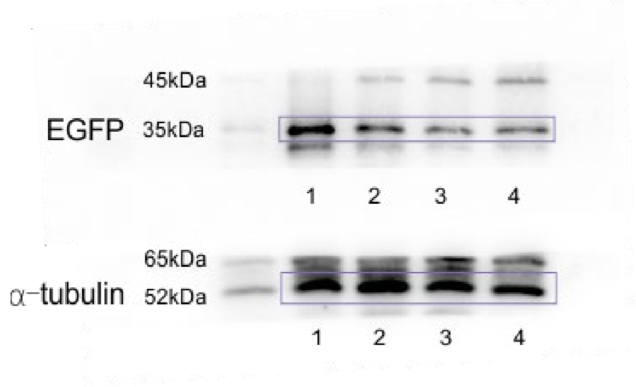

Reference

1. Davison AJ, Akter P, Cunningham C, Dolan A, Addison C, Dargan DJ, et al. Homology between the human cytomegalovirus PL11 gene family and human adenovirus E3 genes. Journal of General Virology. 2003;84:657-63.
2. Kumar MA, Christensen K, Woods B, Dettlaff A, Perley D, Scheidegger A, et al. Nucleosome positioning in the regulatory region of SV40 chromatin correlates with the activation and repression of early and late transcription during infection. Virology. 2017;503:62-9.
3. Kunkel GR, Pederson T. UPSTREAM ELEMENTS REQUIRED FOR EFFICIENT TRANSCRIPTION OF A HUMAN U6 RNA GENE RESEMBLE THOSE OF U1 AND U2 GENES EVEN THOUGH A DIFFERENT POLYMERASE IS USED. Genes & Development. 1988;2(2):196-204.
4. Altman S, Kirsebom L, Talbot S. RECENT STUDIES OF RIBONUCLEASE-P. Faseb Journal. 1993;7(1):7-14.
5. Paige JS, Wu KY, Jaffrey SR. RNA Mimics of Green Fluorescent Protein. Science. 2011;333(6042):642-6.
6. Song W, Strack RL, Svensen N, Jaffrey SR. Plug-and-Play Fluorophores Extend the Spectral Properties of Spinach. Journal of the American Chemical Society. 2014;136(4):1198-201.
